# Colocalization of Erythrocytes and Vascular Calcification in Human Atherosclerosis: A Systematic Histomorphometric Analysis

**DOI:** 10.1055/s-0041-1725042

**Published:** 2021-04-14

**Authors:** Elsa Wilma Böhm, Maria Pavlaki, Georgios Chalikias, Dimitrios Mikroulis, George S. Georgiadis, Dimitrios N. Tziakas, Stavros Konstantinides, Katrin Schäfer

**Affiliations:** 1 Department of Cardiology, University Medical Center, Mainz, Germany; 2 Department of Cardiology, Democritus University of Thrace, Alexandroupolis, Greece; 3 Department of Cardiothoracic Surgery, Democritus University of Thrace, Alexandroupolis, Greece; 4 Department of Vascular Surgery, Democritus University of Thrace, Alexandroupolis, Greece

**Keywords:** atherosclerosis, calcification, erythrocytes, hemoglobin, human pathology

## Abstract

**Background** Intimal calcification typically develops in advanced atherosclerosis, and microcalcification may promote plaque progression and instability. Conversely, intraplaque hemorrhage and erythrocyte extravasation may stimulate osteoblastic differentiation and intralesional calcium phosphate deposition. The presence of erythrocytes and their main cellular components (membranes, hemoglobin, and iron) and colocalization with calcification has never been systematically studied.

**Methods and Results** We examined three types of diseased vascular tissue specimens, namely, degenerative aortic valve stenosis ( *n* = 46), atherosclerotic carotid artery plaques ( *n* = 9), and abdominal aortic aneurysms ( *n* = 14). Biomaterial was obtained from symptomatic patients undergoing elective aortic valve replacement, carotid artery endatherectomy, or aortic aneurysm repair, respectively. Serial sections were stained using Masson–Goldner trichrome, Alizarin red S, and Perl's iron stain to visualize erythrocytes, extracelluar matrix and osteoid, calcium phosphate deposition, or the presence of iron and hemosiderin, respectively. Immunohistochemistry was employed to detect erythrocyte membranes (CD235a), hemoglobin or the hemoglobin scavenger receptor (CD163), endothelial cells (CD31), myofibroblasts (SMA), mesenchymal cells (osteopontin), or osteoblasts (periostin). Our analyses revealed a varying degree of intraplaque hemorrhage and that the majority of extravasated erythrocytes were lysed. Osteoid and calcifications also were frequently present, and erythrocyte membranes were significantly more prevalent in areas with calcification. Areas with extravasated erythrocytes frequently contained CD163-positive cells, although calcification also occurred in areas without CD163 immunosignals.

**Conclusion** Our findings underline the presence of extravasated erythrocytes and their membranes in different types of vascular lesions, and their association with areas of calcification suggests an active role of erythrocytes in vascular disease processes.

## Introduction

Intimal calcification is a typical component of advanced atherosclerotic lesions and particularly common at the aorta, aortic valve, and coronary arteries. 1 The deposition of hydroxyapatite crystals within atherosclerotic lesions is regulated by various molecular mechanisms, which are similar to osteogenesis in bones, and biomarkers for osteoblastic cell differentiation, such as alkaline phosphatase, bone sialoprotein, osteopontin, osteocalcin, collagen II or Cbfa1, have been detected in atherosclerotic lesions of patients 2 3 and aged apolipoprotein-E (ApoE)-deficient mice. 4 However, the relevance of vascular calcification for plaque instability and the occurrence of thrombotic events following plaque rupture is controversial. In this regard, a distinction is made between micro- and macrocalcification: microcalcifications, especially within the fibrous cap, are considered destabilizing, 5 whereas macrocalcifications are believed to protect against rupture. 6

Another phenomenon of advanced atherosclerotic lesions is neovascularization which occurs in response to angiogenic cues generated during local hypoxia as a result of plaque growth. Newly formed vessels within lesions are often immature and leaky and may be the source of local bleeding, so-called intraplaque hemorrhage (IPH). 7 In accordance, extravasation of erythrocytes and their components is frequently observed in atherosclerotic lesions. 8 Interestingly, clinical and experimental studies of coronary arteries, 9 carotid plaques 10 and degenerated aortic valves 11 have shown that intraplaque hemorrhage is associated with the progression of vascular and valvular disease. In this context, we and others have previously shown that cholesterol and other lipids in the erythrocyte membrane promote the expansion of the lipid plaque core. 12 13 14 Chemokines bound to the nonsignaling Duffy's antigen receptor for chemokines (DARC) on the erythrocyte surface may locally deposit proatherosclerotic mediators and promote inflammatory cell recruitment and inflammation. 15 16 17 18 Recently, we could show that lysed but not intact erythrocytes cause osteogenic differentiation of vascular smooth muscle cells (SMCs) in vitro and ex vivo and promote calcification of preexisting vascular lesions in mice. 19

Based on these previous findings, the aim of this study was to systematically analyze the presence of erythrocytes and their components in different types of human vascular and valvular lesions, namely, stenotic aortic valves, abdominal aortic aneurysms, and atherosclerotic carotid plaques. Histological and immunohistochemical stainings were employed on serial sections through these lesions to visualize the colocalization of erythrocyte components and biomarkers of hemolysis or vascular calcification. By histologically demonstrating a remarkable degree of colocalization of erythrocytes and calcification in the vascular wall, indications of possible molecular connections could be found. Overall, our observations support the concept that erythrocytes not only contribute to the progression of atherosclerosis but also to the development of vascular and valvular calcification.

## Materials and Methods

### Reagents

The chemicals and solutions used for tissue fixation, histology, and immunohistochemistry were purchased from Applichem (acetone, catalogue number A1582; ethylenediaminetetra-acetic acid, catalogue number A3553), Carl Roth (Alizarin red S or Alizarin sulphonic acid sodium salt; catalogue number 0348.2; ethanol, catalogue number K9285; formaldehyde solution 37%, catalogue number 4979.1; glacial acetic acid, catalogue number 3738.4; hydrogen peroxide, catalogue number 9681.4; molybdatophosphoric acid hydrate, catalogue number 4440.3; nuclear fast red aluminum sulfate solution, catalogue number N069.1; Orange G, catalogue number 0318.2; Roticlear, catalogue number A538.1; Weigert's hematoxylin solution A and B, catalogue numbers X906.1 and X907.1; xylene, catalogue number 9713.5), Dako (antibody diluent, background reducing, S302283–2), Sigma-Aldrich (acid fuchsin, catalogue number F8129; citric acid monohydrate, catalogue number C1909; ponceau xylidine, catalogue number P2395; potassium hexacyanoferrate, catalogue number P3289; saturated picric acid solution, catalogue number P6744; and zinc formaline, catalogue number Z2902–3), Vector Laboratories (AEC Peroxidase Substrate Kit, catalogue number SK-4200; Vectastain Elite ABC-HRP kit, catalogue number PK-6100) or Waldeck GmbH (azophloxine, catalogue number 1B-103; light green SF, catalogue number 1B-211R).

### Human Vascular and Valvular Tissue Specimens

Tissue specimens of degenerative aortic valves, carotid artery plaques, and abdominal aortic aneurysms were obtained from symptomatic patients undergoing elective surgery at the University General Hospital of Alexandroupolis, Greece, Departments of Cardiothoracic Surgery and Vascular Surgery, respectively. Exclusion criteria were active cancer, systemic inflammatory, or autoimmune disease. Tissue specimens were stored in sterile physiological saline solution (0.9% NaCl) on ice, transferred to our laboratory, and immediately processed for paraffin embedding, including fixation for 24 hours at 4°C in zinc formaline solution followed by storage in 70% ethanol until paraffin embedding (within a week).

The study protocol complied with the declaration of Helsinki and was approved by the institutional review board (University Hospital Alexandroupolis, Greece; protocol number 561/10–06–2013). All participants were informed and gave their signed consent prior to inclusion in the study.

### Histological and Immunohistochemical Analyses

Paraffin-embedded vascular tissues were decalcified by incubation in 10% EDTA (pH = 7.4) overnight and serially cut into 10 5-µm thick cross-sections. After being postfixed overnight under the hood in Bouin's fixative (750 ml saturated picric acid solutuion, 250 ml 37% formaldehyde solution and 50 ml glacial acetic acid), section number one was stained using Masson–Goldner trichrome to detect erythrocytes, connective tissue, osteoid, and bone. Calcium phosphate depositions were visualized on section number two by staining with 0.5% Alizarin red S, iron, and hemosiderin on section number three using Perl's iron stain.

Immunohistochemical analyses were performed on sections number 4 to 10 using monoclonal mouse anti-human antibodies against CD163 (abcam; catalogue number ab111250; dilution, 1:25), CD235a (Dako; catalogue number M081901–2; dilution, 1:200), CD31 (Dako; catalogue number M082301–2; dilution, 1:50) and myofibroblasts (SMA; Sigma; catalogue number A2547; dilution, 1:500), or polyclonal rabbit anti-human antibodies against hemoglobin (Abcam; catalogue number ab191183; dilution, 1:250), osteopontin (abcam; catalogue number ab8448; dilution, 1:500), and periostin (ThermoFisher Scientific; catalogue number PA5–34641; dilution, 1:50). Sections were deparaffinized in xylole and a series of graded ethanol (100, 96, 70, and 50%). Heat-induced epitope retrieval was performed in 0.01-M citrate buffer (pH = 6.0) for 11 minutes at 800 W, using a microwave oven. Unspecific binding sites were blocked by incubation with 10% normal goat serum (abcam, catalogue number: ab156046). Following overnight incubation at 4°C in a humid chamber, slides were incubated with biotinylated secondary goat anti-mouse (Molecular Probes; catalogue number B2763; dilution, 1:1.000) or goat antirabbit (Molecular Probes; catalogue number B2770; dilution, 1:1.000) antibodies for 60 minutes at room temperature. After three washes for 5 minutes each in phosphate-buffered saline (PBS), avidin–biotin horseradish peroxidase link was added for 30 minutes followed by incubation with freshly prepared substrate solution (3-amino-9-ethylcarbazole or AEC) until color development. Sections were briefly counterstained with Gill's hematoxyline before being mounted with aqueous mounting medium (ImmuMount; Thermo Fisher Scientific; catalogue number 9990402). For each experiment, a positive (human tissue containing the antigen of interest, i.e., liver for CD163; lung for CD235a and hemoglobin; kidney for CD31; artery for SMA, bone for osteopontin and periostin) and negative (omission of the first antibody) controls were examined in parallel.

### Morphometric Analysis

Sections were examined under an Olympus BX51 microscope and completely photographed at different magnifications (40-, 200-, 400-, and 1,000-fold magnifications) using the MicroPublisher 5.0 RTV cooled color digital camera (QImaging, Surrey, BC, Canada). For the quantitative and qualitative analyses of the histochemical stainings, samples (aortic valve samples: *n* = 46, carotid artery: *n* = 9, and aortic aneurysm: *n* = 14) were graded in the categories “negative,” “ + ,” “ + +,” and “ + + + ” based on the amount of the structure of interest, that is, erythrocytes and osteoid after Masson–Goldner trichrome staining, calcification after Alizarin red S staining, and hemosiderin after Perl's iron staining. For the analysis of immunohistochemical stainings and the analysis of colocalizations, the number of viewing fields containing the signal of interest in relation to all viewing fields was determined. For the analysis of colocalization, all areas containing erythrocytes (CD235a-positive cells) were photographed and the presence or absence of calcification was documented on corresponding areas within neighboring sections stained with Alizarin red S and/or antibodies against the osteoblast marker periostin. All quantitative analyses were performed using the image analysis software (Image-Pro Plus; Media Cybernetics).

### Statistical Analysis

Data are shown total or relative numbers (%). Categorical parameters were compared using *χ* ^2^ test. All analyses were performed using GraphPad Prism version 8.0.0 for Windows (GraphPad Software, San Diego, California United States; www.graphpad.com ).

## Results

Previous studies reported the presence of erythrocytes in human and experimental atherosclerotic lesions; however, the concept that erythrocytes may play an active role in the pathogenesis of atherosclerosis has only recently emerged. We showed that membrane components of lysed erythrocytes promote the osteoblastic differentiation of SMCs in culture and vascular calcification of murine aortic rings and established vascular lesions. 19 Here, we systematically examined the presence of erythrocytes and their main components (membranes, iron, and hemoglobin) in human biomaterial obtained from patients with advanced atherosclerosis. For this purpose, we examined a total of 69 human valvular and vascular tissue samples derived from stenotic aortic valves ( *n* = 46), atherosclerotic carotid arteries ( *n* = 9), or abdominal aortic aneurysms ( *n* = 14). Samples were obtained from patients undergoing aortic valve replacement, carotid artery, or aortic aneurysm repair, respectively, at the University Hospital Alexandroupolis, Greece.

### Presence of Erythrocytes in Human Vascular and Valvular Lesions

**Fig. 1:**
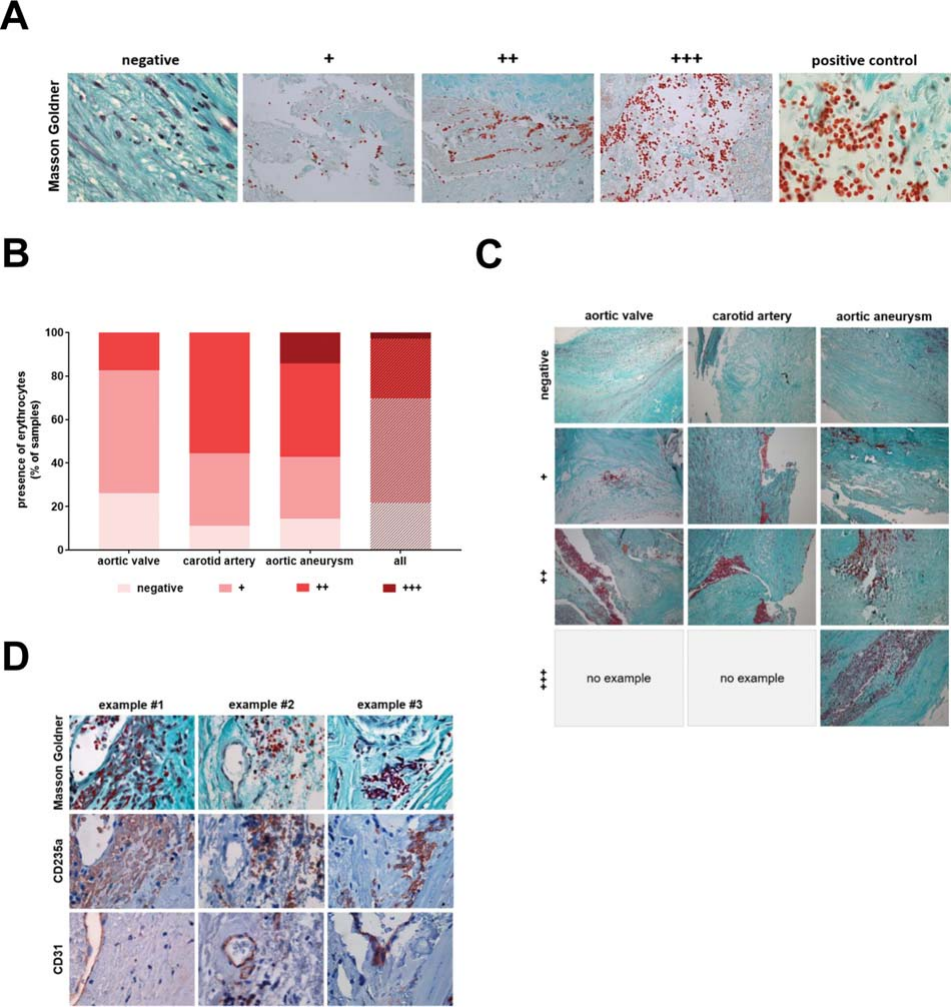
Detection of erythrocytes in human vascular lesions. ( **A** ) Examples of the extent of erythrocyte extravasation and its grading in absent (negative), low (+), middle (++) and high (++ + ). The positive control shows a higher magnification of the typical erythrocyte shape (bright red signal), the example for a negative result shows smooth muscle cells/myofibroblasts in pink, whereas bright red erythrocytes are absent; 1,000-fold (negative and positive control) and 200-fold (all others) magnifications. ( **B** ) Results of the quantitative analysis. Results are expressed as percentage (%) of the total number of tissue samples. ( **C** ) Representative findings of erythrocytes in samples of human aortic valve, carotid artery plaque and aortic aneurysm; 200-fold magnification. ( **D** ) Three examples to demonstrate to localization of erythrocytes following Masson–Goldner trichrome staining (top row) or CD235a immunohistochemistry (middle row) in relation to intralesional CD31-immunopositive endothelial cells lining vascular structures are shown; 1,000-fold magnification.

First, and to obtain a histological overview of the quality of the tissues and their cellular and structural compositions, serial cross-sections through vascular lesions were stained using Masson–Goldner trichrome to visualize erythrocytes (bright red), connective tissue, and acidic mucus substances (pale green), cytoplasm (pale red), as well as cell nuclei (black). Erythrocytes were detected in 34 out of 46 aortic valve samples (73.9%), 8 out of 9 carotid artery plaque samples (88.9%), and 12 out of 14 aortic aneurysm samples (85.7%). Semiquantitative grading of the presence of erythrocytes in vascular lesions using the scale shown in Fig. 1A revealed the highest degree (++ + ) of erythrocytes in aortic aneurysms (14.3%), whereas the percentage of tissue samples negative for erythrocytes was highest in the aortic valve (26.1%). The results of this analysis are shown in Fig. 1B , representative examples in Fig. 1C . Findings after Masson–Goldner trichrome staining ( Fig. 1D , top row) were confirmed following immunohistochemical detection of the human erythrocyte surface antigen glycophorin A (CD235a) on neighboring sections ( Fig. 1D , middle row). Immunohistochemical staining of endothelial cells in a subset of samples using antibodies against CD31 and microscopical evaluation at higher magnification revealed that erythrocytes, if present, were observed outside the lumen of intraplaque vessels in the majority of all viewing fields containing erythrocytes ( Fig. 1D , bottom row), that is, in 83.3% (10 out of 12 viewing fields) in aortic valve samples, 90.9% (10 out of 11 viewing fields) in carotid artery plaque samples and 84.1% (16 out of 19 viewing fields) in aortic aneurysm samples.

### Presence of Calcification in Human Vascular and Valvular Lesions

**Fig. 2:**
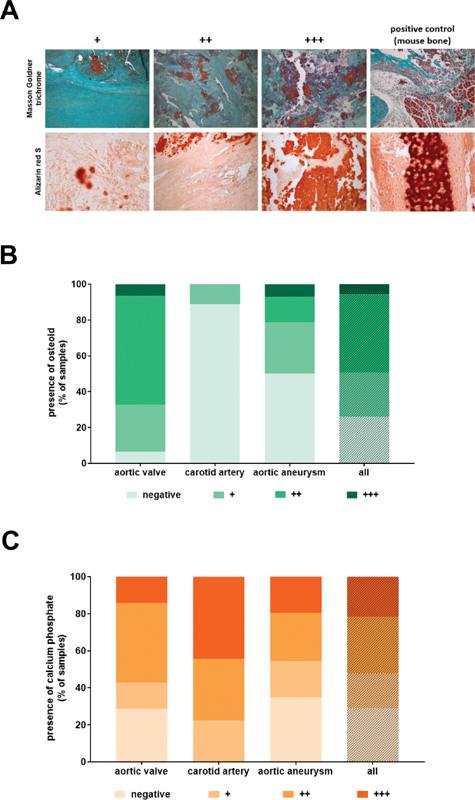
Detection of calcification in human vascular lesions. ( **A** ) Examples of histochemical stainings and their grading in absent (negative), low (+), middle (++) and high (++ + ). Findings in mouse bone (positive control) to demonstrate typical positive signals; 200-fold magnification. ( **B** ) Results of the quantitative analysis after Masson–Goldner trichrome staining. ( **C** ) Results of the quantitative analysis after Alizarin red S staining. Results in ( **B** ) and ( **C** ) are expressed as percentage of the total number of tissue samples.

Histochemical staining of serial cross-sections and analysis of vascular and valvular calcification using Alizarin red S to visualize calcium phosphate depositions (red signal) and comparison with results after Masson–Goldner trichrome stain to discriminate osteoid (red signal) from mature bone matrix (dark green signal) revealed the presence of both early (osteoid) and advanced (calcium phosphate) forms of osteogenesis in all of the examined vascular lesion types. Representative histological findings and the results of the quantitative analysis of all tissue samples are shown in ( Fig. 2 ). Immunohistochemical detection of the extracellular matrix protein osteopontin, the mesenchymal/smooth muscle cell marker smooth muscle *α* -actin or the osteoblast marker periostin on serial sections revealed colocalization with Alizarin S in 51.9, 72.2, or 96.3% of the analyzed microscopic viewing fields (periostin and osteopontin: *n* = 27; SMA: *n* = 18), respectively (not shown). The distribution patterns for the different degrees of calcification were very similar with that of erythrocytes, particularly in aortic aneurysm samples, whereas large amounts of connective tissue and acidic mucus substances (green signal) were observed at the carotid artery.

### Colocalization of Erythrocyte Membranes and Calcification

Analysis of Masson–Goldner trichrome and Alizarin red S-stained vascular tissue sections revealed the presence of calcification in 87.4% of all areas showing erythrocytes, and erythrocytes in 70.4% of all areas with calcification (not shown). More specifically, calcification was observed in 92.0% of all microscope viewing fields containing erythrocytes in aortic valve specimens, 86.7% in carotid artery samples, and 84.4% in aortic aneurysm ( Table 1 ). To further examine the possible association of intraplaque hemorrhage, erythrocyte lysis and calcification, we used CD235a antigen as marker to visualize erythrocyte membrane deposition and examined its colocalization with areas of calcification, that is, showing Alizarin red S and/or periostin positive signals. The results after analysis of a total of 87 microscope fields in human samples of all three lesions types (aortic valve stenosis, carotid artery plaque, and aortic aneurysm) are given in Table 2 . They show that areas with calcification are detected with a significantly higher frequency in areas containing CD235a-positive erythrocyte membranes compared with CD235a-negative areas.

**Table 1:** Frequency of calcification in areas with erythrocytes within human valvular and vascular lesions

	Erythrocytes	Colocalization with calcification	Percentage (%)
Aortic valve	25	23	92.0
Carotid artery	30	26	86.7
Aortic aneurysm	32	27	84.4
Total	87	76	87.4

Note: Data show the number of microscopic viewing fields containing areas with calcification as percentage (%) of all viewing fields containing erythrocytes (Masson–Goldner trichrome stain).

**Table 2:** Presence of calcification and its dependence of erythrocyte in all types of vascular lesions

	Calcification	No calcification	*p* -Value
CD235a positive	54	6	<0.0001 a
CD235a negative	8	19	

Note: Data refer to microscopic viewing fields and are shown as absolute numbers.

a Determined using *χ* ^2^ test.

**Fig. 3:**
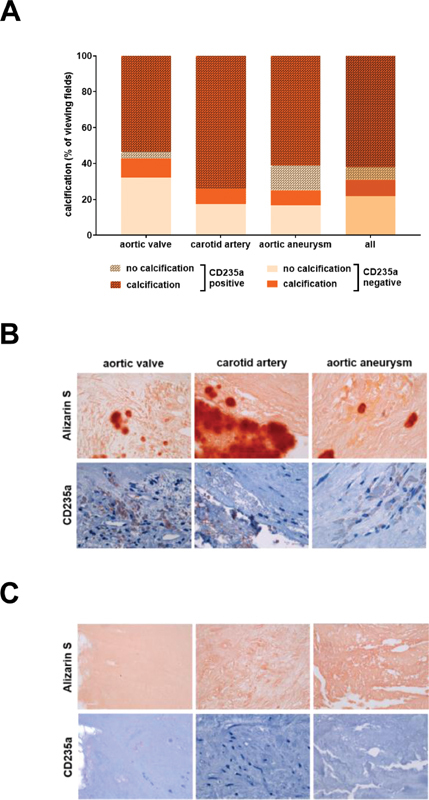
Colocalization of erythrocyte membrane markers and areas with or without calcification in human vascular lesions. ( **A** ) Results of the quantitative analysis. Results are expressed as percentage (%) of the total number of viewing fields. Representative findings showing examples for the colocalization of CD235a and Alizarin red S ( **B** ) or the absence of calcification in areas not containing erythrocyte membranes in samples of human aortic valve stenosis, carotid artery plaque and aortic aneurysm ( **C** ); 1,000-fold magnification.

Separate analysis of the colocalization of CD235a-positive ( Table 3 ) or CD235a-negative ( Table 4 ) areas and areas with calcification in the different lesion types confirmed this observation and showed that calcification is observed with higher frequency in areas containing erythrocyte membranes compared with those without, as observed at the aortic valve (93.8 vs. 25%), carotid artery (100 vs. 33.3%), and aortic aneurysm (81.5 vs. 33.3%). These findings are also presented in Fig. 3 showing that areas simultaneously containing erythrocyte membranes (as indicated by the presence of CD235a antigen) and calcification (as indicated by the presence of Alizarin red S-positive material and/or periostin-positive signals) were most common in all vascular tissue types, followed by those containing neither erythrocyte membranes nor calcification. On the other hand, areas with calcification not containing CD235a or areas containing erythrocyte membranes but staining negative for calcification markers were less frequently observed. The summary of the quantitative analysis is shown in Fig. 3A , representative findings in Fig. 3B and C .

**Table 3:** Calcification in the presence of CD235a-positive erythrocytes

	CD235a positive	Colocalization with calcification	Percentage (%)
Aortic valve	16	15	93.8
Carotid artery	17	17	100
Aortic aneurysm	27	22	81.5
Total	60	54	90.0

Note: Data show the number of microscopic viewing fields containing areas with calcification as percentage (%) of all viewing fields containing CD235a-positive immunosignals.

**Table 4:** Calcification in the absence of CD235a-positive erythrocytes

	CD235a negative	Colocalization with calcification	Percentage (%)
Aortic valve	12	3	25.0
Carotid artery	6	2	33.3
Aortic aneurysm	9	3	33.3
Total	27	8	29.6

Note: Data show the number of microscopic viewing fields containing areas with calcification as percentage (%) of all viewing fields not containing CD235a-positive immunosignals.

### Colocalization of Iron, Extracellular Hemoglobin, and Calcification

**Fig. 4:**
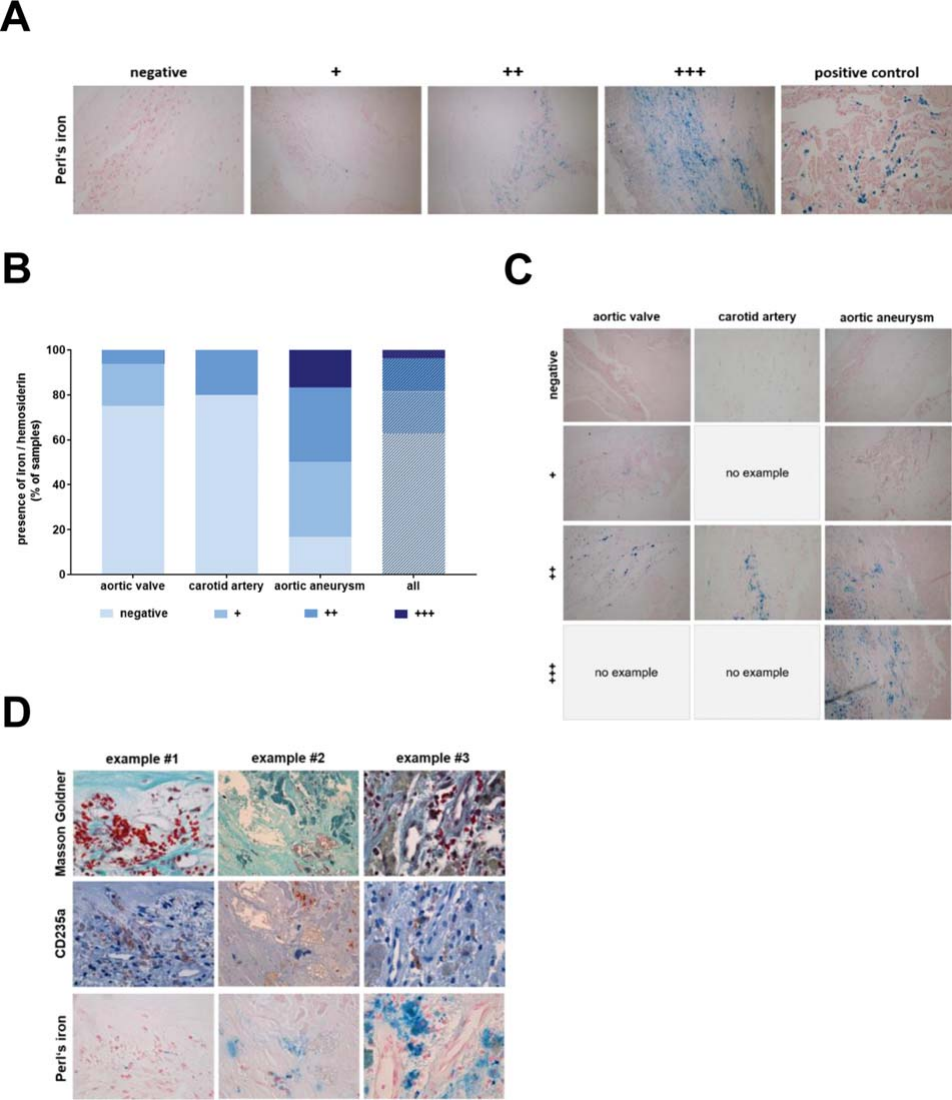
Detection of free iron and hemosiderin in human vascular lesions. ( **A** ) Examples of the extent of iron/hemosiderin deposition and its grading in absent (negative), low (+), middle (++) and high (++ + ); 200-fold magnification. Findings in murine lung (positive control) are shown to demonstrate typical positive signals. ( **B** ) Results of the quantitative analysis. Results are expressed as percentage (%) of the total number of tissue samples. ( **C** ) Representative findings of iron/hemosiderin depositions in samples of human aortic valve, carotid artery plaque and aortic aneurysm; 200-fold magnification. ( **D** ) Examples showing the colocalization of erythrocytes (following Masson–Goldner trichrome or CD235a staining) with iron/hemosiderin following Perl's iron staining. 1,000-fold magnification.

**Fig. 5:**
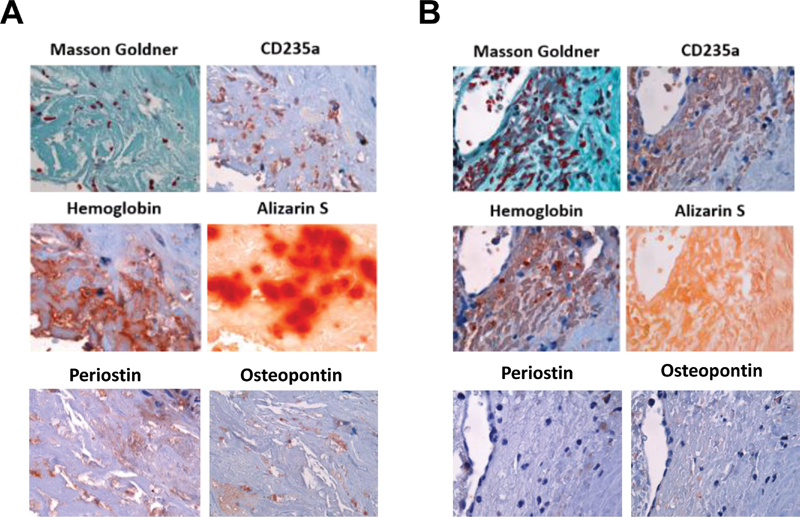
Colocalization of lysed and intact erythrocytes and hemoglobin with areas of calcification in serial sections of human vascular lesions. ( **A** ) Representative example showing the presence of lysed erythrocytes and extracellular hemoglobin in close association with Alizarin S-positive material. ( **B** ) Representative example showing the presence of intact erythrocytes and cell-associated hemoglobin in an area without calcification; 1,000-fold magnification.

Analysis of tissue samples for the presence of hemolysis using Perl's iron stain to detect free iron or its storage form hemosiderin revealed a similar pattern, in agreement with the notion that the majority of intraplaque erythrocytes are not intact ( Fig. 4 ). The above findings suggested that erythrocyte extravasation and lysis frequently occur in human vascular lesions and that these events are associated with calcification. In addition to iron and its storage forms, erythrocyte lysis liberates hemoglobin, the main cytoplasm protein component of erythrocytes. To begin to determine the possible association of hemoglobin with calcification, immunohistochemical detection of CD235a and hemoglobin in vascular lesions was performed. Representative examples showing lysed erythrocytes (defined by the loss of their typical shape after Masson–Goldner trichrome stain, an irregular, spotted pattern of CD235a and confluent, extracellular pools of hemoglobin immunosignals) colocalizing with areas of calcification are shown in Fig. 5A , representative examples showing intact erythrocytes (defined by their typical shape after Masson–Goldner trichrome stain, membrane localization of CD235 immunosignals, and single, cell-associated hemoglobin immunosignals) with areas without calcification are shown in Fig. 5B . The results of the quantitative analysis of the presence of lysed erythrocytes in microscope viewing fields, separately for each of the examined three types of human vascular lesions, are shown in Table 5 . These analyses revealed that the majority of erythrocytes detected in human vascular lesions was lysed, that is, in 53 out of 59 (89.8%) of the viewing fields selected for this analysis.

**Table 5:** Frequency of erythrocyte lysis in human valvular and vascular lesions

	Lysed erythrocytes	Erythrocytes	Percentage (%)
Aortic valve	18	19	94.7
Carotid artery	8	10	80.0
Aortic aneurysm	27	30	90.0
Total	53	59	89.8

Note: Data show the number of viewing fields containing lysed erythrocytes as percentage (%) of all viewing fields containing erythrocytes.

### Presence of Hemoglobin and the Hemoglobin Scavenger CD163 in Areas with Calcification

**Fig. 6:**
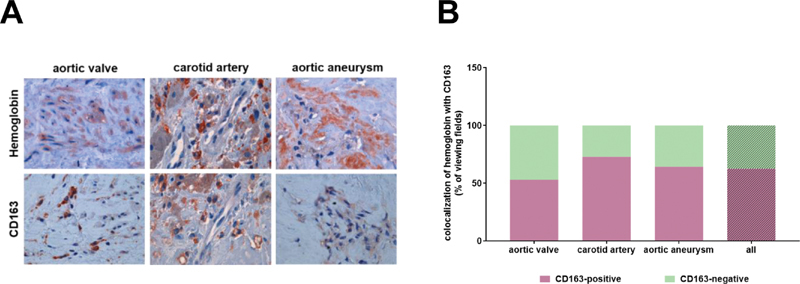
Colocalization of hemoglobin with the hemoglobin receptor CD163 in human vascular and valvular tissue specimens. ( **A** ) Representative examples of findings in the aortic valve, carotid artery and aortic aneurysm are shown; 1,000-fold magnification. ( **B** ) Results of the quantitative analysis of hemoglobin-positive areas and their colocalization with CD163 immunosignals. Results are expressed as percentage (%) of the total number of viewing fields.

**Fig. 7:**
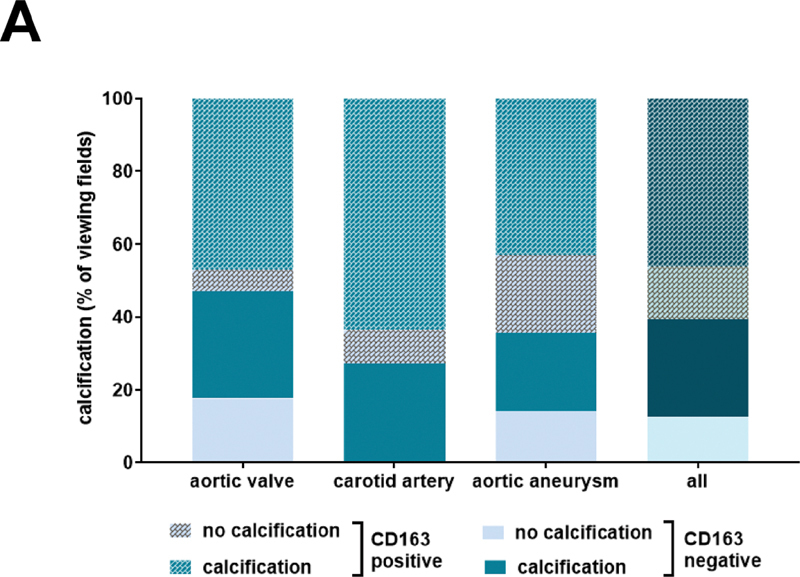
Colocalization of the hemoglobin scavenger receptor CD163 with calcification in human vascular lesions. ( **A** ) Results of the quantitative analysis of the colocalization of CD163 and Alizarin S in samples of human stenotic aortic valves, carotid artery plaques and aortic aneurysms. Results are expressed as percentage (%) of the total number of viewing fields.

Extravascular hemoglobin is removed by the scavenger receptor CD163, 20 exclusively expressed on cells of the monocyte-macrophage lineage. 21 Representative examples showing the colocalization of hemoglobin with the hemoglobin receptor CD163 in human vascular tissue specimens from the aortic valve, carotid artery, and aortic aneurysm are shown in Fig. 6A . Quantitative analysis revealed their simultaneous presence in more than 50% of all microscopic viewing fields in vascular specimens ( Fig. 6B ). We next quantified the percentage of areas within human vascular lesions positive or negative for the hemoglobin receptor CD163 and association with the presence of calcification (Alizarin red S-positive and/or periostin-positive; Table 6 ). Although CD163-positive areas were more often associated with calcification than with no calcification, calcified areas were also frequently negative for CD163, and the statistical analyses did not detect a significant association. The results of these analyses separately for each vascular lesion type are also presented in Fig. 7A . Calcification was most frequently observed in CD163-immunopositive areas in stenotic aortic valves, carotid artery plaques, and aortic aneurysms, whereas CD163-negative areas were to a much lesser extent found to also be calcified. The results of the analysis of the combined presence of hemoglobin and CD163 in areas with calcification are summarized in Table 7 . These analyses revealed that hemoglobin is frequently accompanied by the presence of CD163 in areas with calcification, suggesting that not the presence of hemoglobin per se, but rather its scavenging and removal play a role in vascular calcification.

**Table 6:** Detection of CD163 in areas with and without calcification in all types of vascular lesions

	Calcification	No calcification	*p* -Value
CD163 positive	27	8	0.3914 a
CD163 negative	14	7	

Note: Data refer to microscopic viewing fields and are shown as absolute numbers.

a Determined using *χ* ^2^ test.

**Table 7:** Presence of hemoglobin and CD163 calcification and its association with calcification

	Hemoglobin + CD163 positive	Colocalization with calcification	Percentage (%)
Aortic valve	9	8	88.9
Carotid artery	8	7	87.5
Aortic aneurysm	18	12	66.7
Total	35	27	77.1

Note: Data show the number of viewing fields containing areas with calcification as percentage (%) of all viewing fields containing hemoglobin- and CD163-positive immunosignals.

## Discussion

Erythrocytes are the most abundant cell type in the circulating blood. Although their main function is to transport oxygen and carbon dioxide between lung, tissues, and cells, they are also involved in several other physiological processes, such as the regulation of vascular tone, blood flow, and hemostasis. Their structure, composition, and high deformability are optimized to fulfil these functions. Hemoglobin, the main cytoplasmic component of erythrocytes, binds and releases O _2_ depending on local tissue oxygen levels. 22 The release and binding of other gaseous transmitters, such as carbon monoxide and nitric oxide, allow them to signal toward endothelial and smooth muscle cells, resulting in hypoxic vasodilation 23 among others. They are also part of blood clots and express receptors that allow them to bind fibrinogen or to interact with platelets and endothelial cells suggesting an active role in thrombus formation and resolution. 24

The presence of erythrocytes and their remnants (i.e., heme, hemoglobin, and iron) within advanced atherosclerotic lesions was described some time ago. 8 9 25 26 While earlier studies interpreted these findings as passive consequence of lesion instability, clinical studies suggested that erythrocyte extravasation and intralesional hemorrhage itself might contribute to the progression of atherosclerosis. 9 Serial high-resolution magnetic resonance imaging revealed that intraplaque hemorrhage accelerated plaque progression possibly by increasing the lipid core and total plaque volume. 10 Analysis of human aortic valve specimens from patients with degenerative aortic valve stenosis detected intraleaflet hemorrhage using antibodies against glycophorin A (CD235a), similar to our study, in 78% of all specimens. 11 Intraplaque hemorrhage was identified as the sole factor independently associated with rapid aortic valve stenosis progression in this study. 11

The accumulation of free cholesterol from erythrocyte membranes and lipid core expansion may promote lesion destabilization, 27 making intraplaque hemorrhage a trigger of plaque vulnerability. 28 Focal microhemorrhage has been found to contribute to lipid accumulation, foam cell formation, and macrophage activation in human atherosclerotic plaques. 29 Intravascular ultrasound analysis of autopsy hearts revealed hemorrhage in 0.8% of all coronary artery segments, with a higher frequency in fibroatheromas and segments with a greater lipid core burden. 30

Similar to our findings, a high degree of hemorrhage and iron deposition was also observed in human and murine abdominal aortic aneurysms, 31 32 a special form of atherosclerotic vascular disease characterized by local wall thinning, outward remodeling, and aortic diameter expansion. 33 Although the main complication and clinical presentation of aortic aneurysm are overt wall rupture and bleeding, altered hemodynamic forces resulting in stasis and clot formation may promote local erythrocyte trapping and hemolysis in earlier, clinically silent stages of the disease. The accumulation of iron with subsequent macrophage activation and recruitment may potentiate aortic wall inflammation, 34 and increased matrix metalloproteases activity may promote hemorrhage. 35

As mentioned above, experimental and clinical evidences suggest that erythrocytes directly act on cells of the vessel wall and actively participate in cardiovascular disease processes. 36 Experimental studies also support the active contribution of erythrocytes to atherosclerosis. For example, reduced atherosclerotic lesion progression was observed in ApoE-knockout mice treated with phenylhydrazine to induce anemia. 37 We and others have shown that the deposition of cholesterol-rich lysed erythrocytes, in particular, and their membrane fraction actively contributes to atherosclerotic lesion growth, both in rabbits 12 and humans. 13 Moreover, administration of lipid-lowering statin therapy to rabbits fed atherogenic diet resulted in reduced erythrocyte cholesterol levels, as well as smaller lipid cores, fewer macrophages, and less microvessels. 12 Interestingly, first data indicate that the composition of erythrocytes differs among individuals with cardiovascular risk factors or overt disease, as shown for the amount of membrane cholesterol. 14 Also, significantly elevated levels of the proangiogenic chemokine interleukin-8 bound to the erythrocyte membrane were observed in patients with acute coronary syndrome and associated with the clinical presentation of coronary artery disease. 38 39

Plaque growth results in local hypoxia which drives local angiogenesis and plaque neovascularization from vasa vasorum. 40 However, these thin-walled neovessels are fragile and immature, so that erythrocytes can extravasate into the plaque in the absence of an overt rupture. 7 In the highly oxidative environment of atherosclerotic lesions, extravasated erythrocytes are rapidly lysed, resulting in the local release of their contents, notably cholesterol, iron, and hemoglobin. The uptake of lipids by macrophages and smooth muscle cells contributes to foam cell formation and lipid core expansion. 29 Cell-free hemoglobin and iron released from lysed erythrocytes and deposited locally may worsen atherosclerosis by increasing the generation of free radicals, oxidative stress, and inflammation, 41 and rapid clearance is essential to prevent its cytotoxic effects. Macrophages express the hemoglobin scavenger receptor CD163 and may phagocytose lysed erythrocyte components, thereby removing iron-containing hemoglobin. 42 Perivascular CD163+ macrophages are enriched in plaque areas containing neovessels and hemorrhage, 11 43 and uptake of hemoglobin via CD163 has been associated with changes toward an anti-inflammatory macrophage phenotype and atheroprotection. 42 44 45 However, opposite findings also have been reported. In human atherosclerotic lesions, CD163-positive macrophages were associated with plaque progression, increased intraplaque angiogenesis, and inflammation. 46 Higher numbers of cells expressing CD163, as well as other M2 macrophage subtype markers, such as CD206 or hemoxygenase-1, were observed in human aortic valve samples with calcification compared with those without pathological calcification. 47 CD163 expression on other cell types potentially involved in vascular remodeling, such as (pulmonary artery) smooth muscle cells, 48 was shown to play a role in the regulation of proliferation. 49

The hemoglobin binding protein haptoglobin is also critical for the removal of cytotoxic cell-free hemoglobin, and a haptoglobin genotype with a reduced antioxidative and anti-inflammatory activity was found to be associated with an increased presence of iron, lipid peroxidation, and macrophage accumulation in apoE −/− mouse plaques. 50

Iron may accelerate atherosclerotic vascular disease by promoting lipid peroxidation, endothelial activation, and inflammation, 51 52 although protective effects of iron have also been reported. 34 ApoE −/− mice carrying a mutated form of the iron exporter ferroportin (resulting in increased blood iron levels) also exhibited increased calcification. 52 Others showed that dietary iron restriction reduces atherosclerosis in mice. 53 Apoptosis of smooth muscle cells in response to the toxic effects of iron may result in less stable, rupture-prone plaques. 54 55

Calcification of atherosclerotic plaques are associated with instability and increased risk of rupture and bleeding. 56 Microcalcifications, especially of the fibrous cap, are an indication of the instability of atherosclerotic plaques. 5 57 Multicontrast magnetic resonance imaging of carotid artery plaques revealed strong and independent correlations of intimal calcification with intraplaque hemorrhage. 58 On the other hand, the relationship may be bidirectional and erythrocyte extravasation and lysis may release disease mediators and also promote calcification. We could recently demonstrate that lysed erythrocyte membranes promote the osteoblastic differentiation of smooth muscle cells in culture and vascular calcification ex vivo and in vivo. 19 Although biomarkers for osteoblastic cell differentiation have been detected in atherosclerotic lesions of patients 2 3 and aged ApoE-deficient mice, 59 the concept that plaque hemorrhage and trapped erythrocytes play an active role during vascular lesion calcification has only recently emerged. The findings shown in this study demonstrate that hemolysis accompanying intraplaque/leaflet hemorrhage is one of the major factors promoting intimal, vascular, and valvular calcification. Others found that exposure of valvular interstitial cells with erythrocytes induced an inflammatory and osteoblastic phenotype and the formation of calcium deposits. 60 Of note, calcification may also occur as a result of necrotic or apoptotic cell death followed by passive mineralization. 61

## Conclusion

Although the presence of erythrocytes and their main cellular components and their association with areas of vascular and valvular calcification have not been systematically analyzed previously, it must be stressed that our ex vivo histological analysis cannot definitely prove causality and that our observations have to be validated in vitro and in animal models. Moreover, biomaterial was obtained from patients undergoing surgical interventions. As a result, only advanced atherosclerotic lesions were studied, whereas earlier disease stages could not be examined. It also must be noted that colocalization studies within areas of calcification is technically challenging due to fragile and brittle nature of the material and the absence of cells in advanced calcified lesions. Finally, time course analyses or the examination of healthy vessels are ethically not acceptable and thus not possible in humans.
